# Suicide among those who use mental health services: Suicide risk factors as evidenced from contact-based characteristics in Victoria

**DOI:** 10.3389/fpsyt.2022.1047894

**Published:** 2022-12-08

**Authors:** Tharanga Fernando, Angela Clapperton, Matthew Spittal, Janneke Berecki-Gisolf

**Affiliations:** ^1^Victorian Injury Surveillance Unit, Monash University Accident Research Centre, Monash University, Clayton, VIC, Australia; ^2^Melbourne School of Population and Global Health, Faculty of Medicine, Dentistry and Health Sciences, The University of Melbourne, Carlton, VIC, Australia

**Keywords:** contact, mental health, service use, suicide, risk factors

## Abstract

**Objective:**

The majority of suicide decedents have had contact with health services in the months before their death. Contacts for mental health services present potential suicide prevention opportunities. This study aims to compare contact-based characteristics among suicide decedents and living controls in the year subsequent to clinical mental health contact with the public health system in Victoria, Australia.

**Methods:**

A population-based nested case-control study of those who had mental health-related hospital and community contacts with the public health system was conducted. Cases (suicide decedents) were age and gender-matched to living controls (suicide non-decedents). These records were linked to records of suicides that occurred in the 12 months following the health service contact, between January 1, 2011, and December 31, 2016. Victorian residents aged 10 years and above were selected at the time of contact (483,933 clients). In the study population, conditional logistic regression models were used to assess the relationship between contact-based characteristics and suicide. Socio-demographics and mental health-related hospital and community contact data was retrieved from the Victorian Admitted Episodes Dataset, the Victorian Emergency Minimum Dataset and the Public Clinical Mental Health database and suicide data from the Victorian Suicide Register.

**Results:**

During a six-year period, 1,091 suicide decedents had at least one mental health contact with the public health system in the 12 months preceding the suicide. Overall, controls used more mental health services than cases; however, cases used more mental health services near the event. The relationship between the type of service and suicide differed by service type: hospital admissions and emergency department presentations had a significant positive association with suicide with an OR of 2.09 (95% CI 1.82–2.40) and OR of 1.13 (95% CI 1.05–1.22), and the effect size increased as the event approached, whereas community contacts had a significant negative association with an OR of 0.93 (95% CI 0.92–0.94), this negative association diminished in magnitude as the event approached (OR∼1).

**Conclusion:**

Suicide decedents had less contact with mental health services than non-decedents; however, evidence suggests suicide decedents reach out to mental health services proximal to suicide. An increase in mental health service contact by an individual could be an indication of suicide risk and therefore an opportunity for intervention. Further, community level contact should be further explored as a possible prevention mechanism considering the majority of suicide decedents do not access the public clinical mental health services.

## Introduction

Suicide is a major public health concern globally, with more than 700,000 suicide deaths occurring worldwide every year (1). According to the World Health Organisation, some of the highest age-standardised suicide rates for 2019 were reported from some African countries, with the highest being 87.5 per 100,000 population in Lesotho, while this rate is estimated to be around 14.5 in the United States, 12.2 in Japan and 11.3 in Australia (2). Suicide has also been identified as the leading cause of age standardised years of life lost in the high income Asia Pacific region and as one of the top 10 leading causes of death in eastern Europe, central Europe, western Europe, central Asia, Australasia, southern Latin America, and high income North America (3, 4). Recent statistics published by the Coroner’s Court of Victoria (CCOV) showed that there were 2,819 suicide deaths in the Australian state of Victoria between 2017 and 2020, an average of 705 suicide deaths a year (5).

Past studies have shown that among those who died by suicide, a majority have had contact with general practitioners (GPs), outpatient and inpatient health services close to the death date (6–11). These contacts can assist in the identification of persons at risk of suicide or assist in providing treatment or other interventions for suicide prevention (7). For example, treatment with medications such as clozapine and lithium have been found to reduce the risk of suicidal behaviour in some populations (12), and active contact and follow-up type interventions have been shown to be effective in preventing repeat suicidal behaviour in patients admitted to EDs for suicide attempts (13).

A systematic review and meta-analysis of relevant studies (most of them carried out in Western European and North American countries) has shown that contact with health services within the year prior to suicide seems to increase; 18.3% of suicide decedents having mental health related contacts with inpatient services, and 26.1% having contact with outpatient mental health services (14). A study conducted in Hong Kong found that only 25% of suicide decedents had received psychiatric care; among these patients, there were more contacts with psychiatrists closer to the suicide date (15). A study conducted in China found that 37% of suicide decedents did not have a diagnosed mental health condition, and among those who did, only 13% received psychiatric treatment, and acute interpersonal crises just prior to the suicide event seemed to contribute to the event (16).

A previous study by us found that of those who died by suicide over the period 2011-2017, 50% had hospital contact for any reason in the 12 months prior to suicide and 28.6% had mental-health-related hospital contact (17).

Multiple US studies have found that suicide decedents had more inpatient admissions and emergency department (ED) presentations compared to controls, for both mental health and non-mental health related conditions (18–20). They also found that closer to the death date, the suicide decedents showed an increase in health care utilisation and that compared to controls, suicide decedents had more healthcare visits, including psychiatric-related contacts, in the last 12 months prior to suicide. They were also more likely to have had both inpatient and outpatient mental health visits; however, ED presentations related to mental health issues were only seen among cases.

The time-dependent relationship between mental health service use and suicide in Australia is not known. We addressed this gap in knowledge by conducting a study of health services data linked with death data. A state-wide data linkage study was undertaken in the Victorian state of Australia, linking mental health-related health service and suicide data, to establish the patterns of service use and suicide risk factors as evidenced from person- and contact-based characteristics. The study had access to ED collected data which does not exist in many other places. This paper constitutes a part of this linkage study with the aim of comparing contact-based characteristics among suicide decedents and living controls in the year subsequent to a mental health related clinical contact with the public health system in Victoria.

## Materials and methods

### Study design

A retrospective analysis of existing morbidity and mortality data in the state of Victoria, Australia was carried out for this study. The study was a population-based nested case-control study of those who used mental health services linked with suicides that occurred in the 12 months following the health service contact.

The study was approved by the Monash University Human Research Ethics Committee (Project no: 14647). A waiver of informed consent was granted on the following grounds: the size of the linked datasets was large and obtaining consent from such a large number of persons was impracticable; the data was de-identified (name, date of birth and other identifiers were removed from the dataset by the Centre of Victorian Data Linkage prior to release of the data to the researchers); the study involvement was low risk for participants’ protection of privacy.

### Data sources and linkage

Mortality data was extracted from the Victorian Suicide Register (VSR) while specific mental health related contact data was extracted from three data sources: namely, the Victorian Admitted Episodes Dataset (VAED), Victorian Emergency Minimum Dataset (VEMD) and the public clinical mental health dataset (Client Management Interface/Operations Data Store).

The VSR is an ongoing register established by the Coroners Court of Victoria (CCOV) and contains information on all suspected and coroner-determined suicides reported to the Victorian Coroners Court (21).

The VAED is a unit record data file of all public and private hospital admissions in Victoria, which consists of patient demographics and morbidity information. Morbidity information includes forty fields related to disease, injury and external causes, coded to the International Statistical Classification of Diseases and Related Health Problems, Tenth Revision, Australian Modification (ICD-10-AM) (22).

The VEMD comprises demographic, administrative and clinical data detailing ED presentations at Victorian public hospitals with designated 24-h emergency departments (currently 39 hospitals). Data are coded to the relevant VEMD User Manual published by the Victorian Department of Health (23).

Specialist mental health services in Victoria are divided into two service delivery types: clinical and non-clinical. Clinical services focus on assessment and treatment of people with a mental illness. The Client Management Interface/Operational Data Store (CMI/ODS) contains data from Victorian Government funded clinical mental health services. CMI/ODS comprises two systems. The Client Management Interface is the local client information system used by public mental health services. The Operational Data Store manages a set of selected data items from each CMI. Data in the CMI includes demographics, clinical status including diagnosis, and service history for each person registered on the CMI/ODS system as receiving public clinical mental health services.

The CCOV supplied the VSR data to the Centre for Victorian Data Linkage (CVDL) at the Victorian Department of Health via secure data exchange portals. CVDL extracted the requested health service contacts data (hospital admissions, ED presentations and outpatient services within the requested time frame) and matched the identifiers in these data extracts with identifiers in the VSR. CVDL then linked the service contact data with the VSR data using these identifiers. Data linkage performed by CVDL used patient-specific identifiers and deterministic data linkage. The de-identified datasets, containing a newly generated, project-specific linkage ID, were transferred to researchers at Monash University using the secure data exchange portal.

### Participants

**FIGURE 1:**
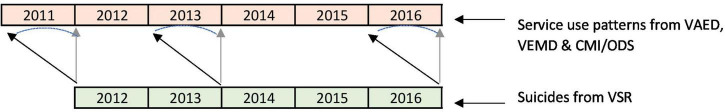
Schematic diagram of the data linkage time periods.

The study population consisted of persons 10 years of age and older, resident in Victoria at the time of contact with the health service, and had a mental health related contact with the public health system in Victoria between 01 January 2011 and 31 December 2016. Cases were extracted from the VSR; initial case selection included all suicide deaths that took place between 01 January 2012 and 31 December 2016. Cases were retained provided they had a mental health related health service contact in the 12 months prior to the suicide date (Figure 1). Persons with sex coded as ‘intersex’ were rare and they were therefore excluded to preserve confidentiality, as well as to avoid inadequate cell counts having an effect on the regression analysis. Persons with no recorded postcode or Local Government Area code for residence were also excluded as this information was required to determine if they were Victorian residents. Contacts for cases with unrealistic time to death, i.e., contact dates occurring after the date of death were further excluded. These unrealistic dates could be due to data quality issues, data linkage inaccuracy or contacts by family members after the death.

**FIGURE 2:**
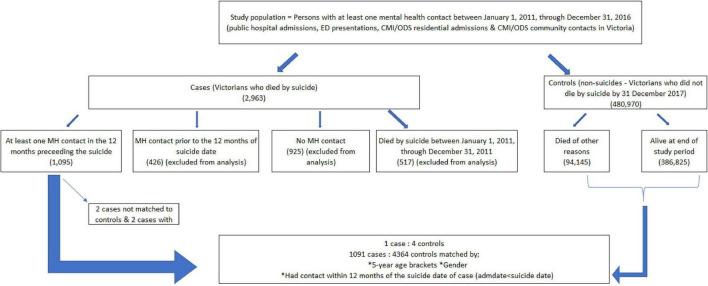
Case and control selection process flow chart.

The total number of suicides in the study period was 2,963, and 1,091 of these decedents had at least one clinical mental health contact with the public health system in the 12 months prior to index date (date of suicide). The number of patients retained for the selection of controls was 480,970. Figure 2 provides a case-control selection flow chart. Cases were selected as people who died by suicide and who had at least one mental health contact in the 12 months prior to the incident. Controls were those who died of other causes or who were alive at the end of the study period. Each case was matched to four controls (*n* = 4364) based on age group and gender, with controls having at least one mental health contact in the 12 months prior to the index date of the case.

### Outcome measure and independent variables (predictors)

The study outcome measure was death by suicide recorded in the VSR. The non-outcome was those who died due to other reasons or were alive at the end of the study period.

#### Selection of mental health service contacts (exposure)

Mental health service contacts were determined from the VEMD, VAED, and CMI/ODS. In the VEMD, this consisted of mental health related ED presentations at VEMD participating hospitals. In the CMI/ODS, this consisted of inpatient care (admissions to wards or residential care) and community contacts (direct with client, indirect with client, and all other). In the VAED, this consisted of mental health related admissions at public hospitals (excluding CMI/ODS client inpatient admissions) (see Supplementary Appendix 1 in online supplement for details including diagnosis codes for mental health related exposure measurement). For data analysis, the visit/contact type information was combined with the frequency of contact, resulting in the number of visits/contacts by service type. The number of visits was further categorised by visit timing, that is: the 3 months before suicide (quarter 1), 4-6 months before (quarter 2), 7-9 months before (quarter 3) and 10-12 months before (quarter 4) as well as the full year (12 months) before the suicide.

#### Independent variables (predictors)

Clients were classified into four age groups (10-24, 25-49, 50-74, > = 75 years). The age group selection was based on defining young persons and older persons as suicide/self-harm rates differ by age group and gender (e.g., young females have highest self-harm rates while older males have highest suicide rates). They are also even in width and make sense given different life stages. They were also classified into two geographic region groups (metropolitan Melbourne & regional/rural Victoria) as a remoteness indicator. Socio economic status was classified as per the Socio-Economic Indexes for Areas (SEIFA) (24). The specific SEIFA used in this study was the Index of Relative Socio-Economic Advantage and Disadvantage; a commonly used socio-economic measure in Australia. Based on the Index of Relative Socio-Economic Advantage and Disadvantage, each local government area is allocated a state decile; decile one indicates relatively the greatest disadvantage and least advantage. For analysis, the ten SEIFA deciles were re-categorised into quintiles for ease of interpretation. Country of birth was categorised as born in Australia vs born in any other country.

### Statistical methods

Descriptive analysis on age, gender, SEIFA, country of birth and geographic region were carried out using proportions with 95% confidence intervals (CI) to compare cases with controls. The type of services used were described using the mean number of contacts (and 95% CI) during the 12-month period. The number of contacts by the timing of the visit (i.e., the four quarters most proximal to the index date) was also described using means.

Conditional logistic regression models were used to assess the factors associated with suicide in the study population. Cases were paired with controls with a 1:4 matching. Age and gender were not adjusted for as the control selection was based on age group and gender. Odds ratios (ORs) with 95% confidence intervals and *p* values were used to assess the significance of the risk associated with suicide and the variable of interest. Effect sizes of 30% (calculated as differences in ORs (OR-1) %) or more are also discussed.

In the event of missing geographical information in a certain database; the missing information could be traced from one of the remaining databases using the data linkage person identifier. If this was also not possible, the cases were excluded (*n* = 3). Information on age, gender was generally well recorded in these databases; no cases with missing data were identified.

All tests were carried out at the 5% level of significance. Stata 16.0 (StataCorp) (25) was used to analyse the data.

## Results

### Overview of study sample (cases only)

There were a total of 2963 suicides recorded in the VSR between January 2012 and December 2016 (Table 1). The study sample, defined as having at least one clinical mental health contact with the public health system in the 12 months prior to suicide, as well as the availability of geographic information etc., consisted of 1091 cases (37% of the total). There were notable differences between the study sample vs. all suicide decedents, in terms of demographics. Based on proportions, among all suicide decedents, 29% were males above 50 years of age; however, among those having at least one mental health related service contact, this demographic comprised just 21% of cases. Conversely, females aged 25-75 years made up 19% of all suicide deaths but 25% of those having at least one mental health related service contact (Table 1).

**TABLE 1:** Total suicide deaths and suicide deaths with at least one mental health related service contact, Victoria 2012-2016.

Age group	All suicide deaths [N (col%)]		Suicide deaths with at least one mental health service contact in the 12 months prior to death date [n (col%)]	
		
	Male	Female	Male	Female
10-24 years	277 (9.3)	108 (3.6)	102 (9.3)	54 (4.9)
25-49 years	1083 (36.6)	322 (10.9)	420 (38.5)	170 (15.6)
50-74 years	671 (22.6)	251 (8.5)	187 (17.1)	95 (8.7)
75 + years	190 (6.4)	61 (2.1)	39 (3.6)	24 (2.2)
Total	2,221	742	748	343
*Total gender (row%*)	*75.0%*	*25.0%*	*68.6%*	*31.4%*
Grand total	2,963		1,091	

### Sociodemographic profile of cases and controls

Table 2 presents a comparison of the proportions of cases and controls (using the patient as the unit of analysis), by age group, gender, SEIFA quintiles, country of birth and geographical region. Since controls were matched to cases by age group and gender, the proportion of patients falling into each age band and gender were similar among cases and controls. The proportion of patients falling into each country of birth group and geographical regions were also similar for both cases and controls (*p* values: 0.771 and 0.518). Average SEIFA (as an indicator of socio-economic status) was not substantially higher or lower in cases vs. controls. Overall, there was no significant difference in the sociodemographic parameters between cases and controls.

**TABLE 2:** Demographic characteristics of cases and controls.

Characteristic	Cases (*n* = 1091)	Controls (*n* = 4364)	*P*-value
	
	*n* (%)		
**Age group**			
10-24 years	156 (14.3)	774 (17.7)	0.008
25-49 years	590 (54.1)	2365 (54.2)	0.953
50-74 years	282 (25.8)	1007 (23.1)	0.061
75 + years	63 (5.8)	218 (5.0)	0.285
**Gender**			
Male	748 (68.6)	2992 (68.6)	1.000
Female	343 (31.4)	1372 (31.4)	1.000
**SEIFA quintiles**			
1st quintile	136 (12.5)	598 (13.7)	0.299
2nd quintile	169 (15.5)	623 (14.3)	0.315
3rd quintile	204 (18.7)	768 (17.6)	0.396
4th quintile	296 (27.1)	1219 (27.9)	0.598
5th quintile	286 (26.2)	1156 (26.5)	0.841
**Country of birth**			
Australia	862 (79.0)	3465 (79.4)	0.771
Other	229 (21.0)	899 (20.6)	0.771
**Geographic region**			
Melbourne metropolitan	759 (69.6)	3083 (70.6)	0.518
Regional/Rural Victoria	332 (30.4)	1281 (29.4)	0.518

Case-control matching was based on five-year age groups and gender. Some controls shift age brackets when the index date comes into consideration. I.e., they are initially identified by the algorithm as a match to the case by age group and sex only; however, when the control’s contact with the health service is traced in relation to the case’s index date, the control’s age bracket could have shifted.

### Contacts profile of cases and controls

An overview of the services used by cases and controls is shown in Table 3. A significantly higher proportion of cases had at least one ED presentation (*p* < 0.001), or one public hospital ward admission (*p* < 0.001) compared to controls, whereas a significantly higher proportion of controls had at least one community contact (*p* < 0.001) or residential admission (*p* < 0.001). There was no significant difference between cases and controls in the mean number of contacts for public hospital admissions (*p* = 0.928) or ED presentations (*p* = 0.511); however, controls had significantly higher mean number of contacts for residential admissions (*p* < 0.001) and community contacts (*p* < 0.001).

**TABLE 3:** Patients with at least one mental health contact by service type.

Characteristic	Cases (*n* = 1091)	Controls (*n* = 4364)	*P*-value
	**Number of people (%)**		
Public hospital admissions	740 (67.8)	2,147 (49.2)	<0.001
ED presentations	762 (69.8)	1,771 (40.6)	<0.001
CMI/ODS residential admissions	58 (5.3)	456 (10.4)	<0.001
CMI/ODS community contacts	729 (66.8)	4,076 (93.4)	<0.001
	**Mean number of contacts (95% CI)**		
Public hospital admissions	1.3 (1.2–1.5)	1.3 (1.2–1.4)	0.928
ED presentations	1.6 (1.3–1.8)	1.5 (1.3–1.6)	0.511
CMI/ODS residential admissions	0.1 (0.0–0.1)	0.2 (0.1–0.2)	<0.001
CMI/ODS community contacts	13.3 (11.8–14.9)	64.5 (61.4–67.7)	<0.001

### Change in service use intensity over the 12-month period for cases and controls

#### Contacts (all services combined)

Overall, controls had higher mental health service use (mean number of contacts in total, i.e., all three services combined) in general (16-18 contacts on average based on the quarter) compared to cases (3-6 contacts on average based on the quarter). Among cases, the mean number of contacts doubled (increased from 3 to 6) when proximal to the index date (Table 4).

**TABLE 4:** Mean number of visits per quarter before the index date for cases and controls.

Months before the index date	Cases		Controls		*P*-value (comparing cases with controls by quarter)
		
	Mean number of visits (95% CI)	*P*-value (compared with cases Q1)	Mean number of visits (95% CI)	*P*-value (compared with controls Q1)	
< = 3 months before death (Q1)	6.4 (5.7-7.1)	–	16.0 (15.0-16.9)	–	<0.001
4-6 months before death (Q2)	4.0 (3.3-4.6)	<0.001	16.7 (15.8-17.5)	0.023	<0.001
7-9 months before death (Q3)	2.9 (2.5-3.3)	<0.001	17.2 (16.4-18.1)	<0.001	<0.001
10-12 months before death (Q4)	3.0 (2.5-3.5)	<0.001	17.6 (16.7-18.5)	<0.001	<0.001

Generally, cases had more contacts in the most proximal quarter to the suicide date compared to the other three quarters. The mean number of contacts in the three most distant quarters (Q2 to Q4) to the index date were significantly lower than the mean number of the most proximal quarter (Q1) for cases (Table 4). For controls, the mean number of contacts in the most proximal quarter (Q1) was statistically significantly lower than the rest of the quarters (Q2 to Q4).

#### Contacts by individual service

The mean number of contacts also varied by type of service (see Supplementary Table 1). Cases had higher mean numbers of contact in the most proximal quarter than controls for public hospital admissions and ED presentations, while controls had significantly higher mean numbers of contacts in all quarters for residential admissions and community contacts.

**FIGURE 3:**
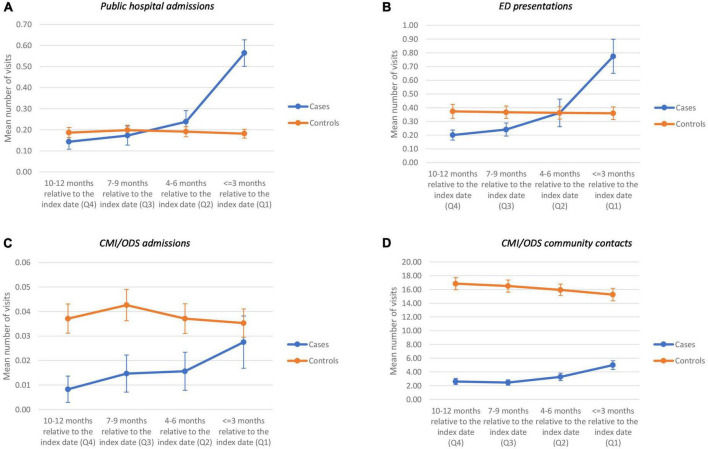
Mean number of visits by quarter in the 12 months prior to suicide for cases and controls by service type **(A–D)**.

**FIGURE 4:**
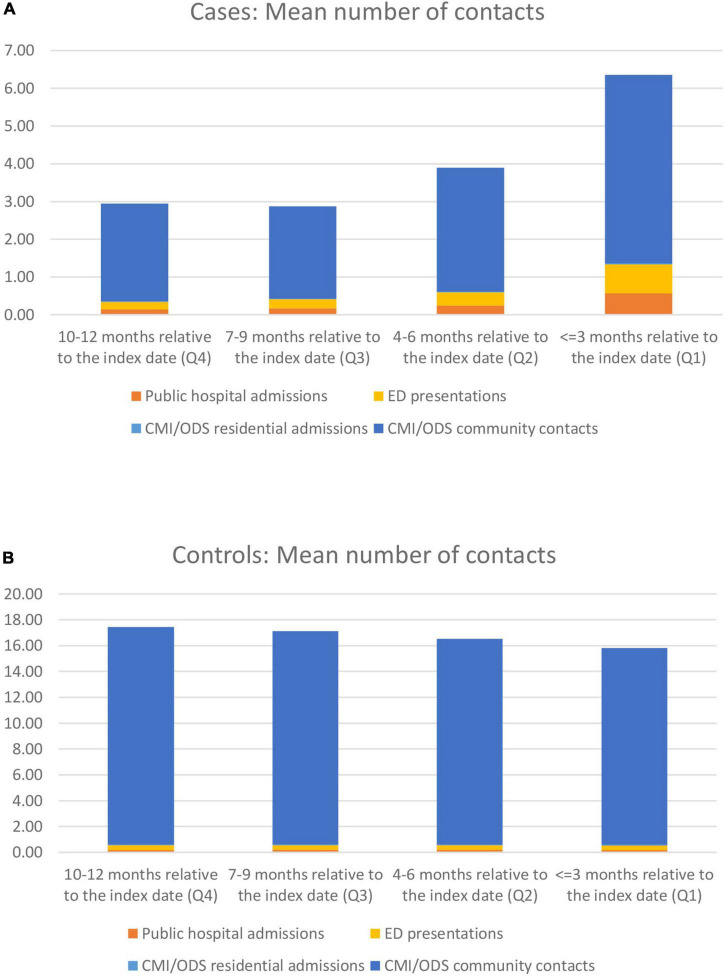
Mean number of contacts by contact type by quarter for cases and controls **(A,B)**.

There was an increase in the mean number of contacts in the most proximal quarter for cases as opposed to controls (which remain quite stable over time, in terms of number of contacts) for all services. This is shown in Figures 3A–D: the increase from the most distant quarter to the most proximal quarter showed a doubling or even greater increase for hospital admissions and ED presentations. Figure 4 summarises the individual results shown in Figure 3. Among cases, the share of public hospital admissions, ED presentations and community contacts increase substantially in the most proximal quarter (Figure 4A); however, there was no such change among controls in the total number of contacts or by service type (Figure 4B).

### Association between the type of service use, timing and number of visits and suicide outcome

**FIGURE 5:**
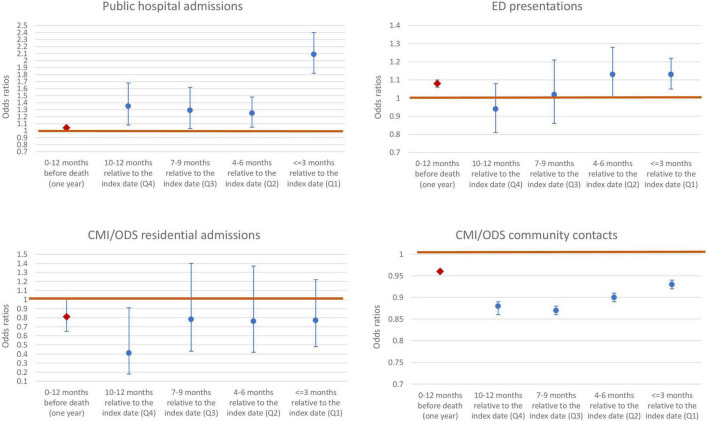
Odds ratios with 95% confidence intervals by contact type.

Conditional logistic regression models were run to assess the association between suicide outcome, and contact frequency by contact type. Figure 5 is a compilation of plots of the ORs for each service by quarter (blue markers) and the entire 12-month period (red markers) (i.e., regardless of the timing) (also see Supplementary Table 2 for details). The conditional logistic model was adjusted for SEIFA quintiles, country of birth and geographical region of residence.

It can be seen that public hospital admissions and ED presentations have a statistically significant positive association with suicide (i.e., OR > 1, indicating an increased likelihood of observing a suicide when the number of contacts increase) (Supplementary Table 2). Residential admissions and community contacts on the other hand have a negative association (OR < 1) indicating a decreased likelihood of observing a suicide when the number of contacts increase.

From Figure 5 it can be seen that the ORs increase as the event gets closer (i.e., towards quarter 1). This further confirms the observation made earlier that the association between service use and suicide (i.e., cases vs. controls) changes over time, leading up to the index date. For some service types, the odds ratios increase closer to the index date (e.g., ED presentations) whereas for other service types, the gap between cases and controls narrows closer to the index date (e.g., community contacts), shown by an attenuation of odds ratios. ED presentations and public hospital admissions have the highest ORs in the most proximal quarter.

## Discussion

This study aimed to identify differences in contact-based characteristics between people who died by suicide who had a clinical mental health related contact with public health services in the 12 months prior to the suicide (cases) and a group of controls matched by age group and gender. Overall, controls used mental health services more than cases; however, proximal to the event, cases showed an increased use of mental health services. For some services, this increase was even to the point of exceeding the average use seen among controls. The strength of the association between contact type and suicide outcome varied by the type of service (contact type).

### Contact profile of cases and controls

Overall, controls had a higher level of mental health service use compared to cases. Controls had approximately 16-18 contacts on average, and this average decreased proximal to the index date. Further analysis showed that this decrease was only significant among the younger age groups (10-24 years) while the older age groups did not show a significant decrease. Possibly, the observed decrease in mental health service contact over time among healthy controls signifies temporary issues in this group, and/or successful resolution of issues. However, among cases, the average number of contacts varied from 3 to 6, and increased proximal to the index date. The average number of contacts varied by type of service as well.

Two studies (18, 19) in the US found that overall, suicide decedents had more health care visits and mental health related visits than controls. This is contrary to what the current study found; out of the four specific mental health services that were included in the study, the number of contacts among cases was not significantly higher than the number among controls. Instead, the mean number of contacts was higher among controls for residential care admissions and clinical mental health related community contacts with public health services. This could be attributed to the fact that the current study is based on those who have had a mental health related health service contact, whilst the US studies are not restricted solely to those with mental health contacts. In other words, the current study explores the association between service use patterns and subsequent suicide, among mental health service users. This is quite different to the analysis of *uptake of mental health services* in the general population, and subsequent suicide. The uptake rate of mental health services by the general population as per the four data sources analysed in the current study was 2.6% in 2016. Our findings that 37% of suicide decedents had accessed mental health services in the preceding twelve months suggest a higher uptake of mental health services in those who died by suicide, *when compared with the general population*. Consistent with the study findings, other studies have also concluded that cases (i.e., those who died by suicide) were more likely to have had more recent contacts with inpatient and community services (14, 15, 18–20).

Among both cases and controls, the community contacts were by far the most highly used service type, in terms of the number of contacts (average of 13.3 among cases and 64.5 among controls). ED presentations were the next most commonly used service (average of 1.6 contacts among cases and 1.5 contacts among controls), followed by public hospital admissions (average of 1.3 among cases and controls). The high use of the community contact service by controls could indicate a “healthy user” practice (26). That is, service use can in some cases be an indicator of pro-active healthy behavior, which is correlated with other healthy practices such as uptake of screening services and healthy lifestyle choices, and therefore also better overall health outcomes.

### Change in service use intensity over the 12-month period for cases and controls

When considering the total number of contacts within the 12 months prior to the index date, a statistically significant positive association between suicide and the number of contacts per service type was seen for public hospital admissions and ED presentations. A statistically significant negative association was seen for community contacts.

The association between the number of contacts and suicide increases in magnitude when closer to the incident for service types that had a positive association (a positive association is seeing an increased likelihood of observing a suicide when the number of contacts increase). In such instances it was seen that this likelihood increases closer to the suicide date. The average use of ED services for cases increased significantly, around three-fold, from the most distant quarter to the quarter most proximal to the index date. Some of these presentations to the ED were for intentional self-harm. These are indicative of cases having more intense interaction with the acute-care health system closer to the suicide date. This finding corroborates conclusions drawn from previous studies (14, 15, 18–20, 27), that closer to the death date, suicide decedents showed an increase in service utilisation. Community contacts were negatively associated with suicide (possibly related to healthy user bias); however, these also showed an increased uptake closer to the index date.

In the quarter most proximal to the index date, the mean number of contacts among cases exceeded the mean number among controls, for ED presentations and public hospital ward admissions. This is only seen in the most proximal quarter. Therefore, mental health related contacts with public hospitals could be a trigger for additional suicide prevention when the same person presents more often than usual.

### Suicide deaths and mental health related contacts with the public health system

Consistent with findings from a previous study (15), a little over one-third of all people who died by suicide in Victoria between 2012 and 2016 had at least one clinical mental health related contact with public health services in the 12 months prior to the suicide. Therefore, the majority of cases *do not access the public clinical mental health services* or have been disconnected with the system in the last 12 months of their life. This in an important point to make, as the study findings only relate to those who have had any mental health contact in the publicly funded system in Victoria.

### Strengths

A key strength of this study is related to the unique findings that differ from the previous literature. Former studies have consistently found that suicide decedents use health care services more so than controls; whereas this study shows that in a mental health service user population, controls used the services more frequently than cases. Notwithstanding this difference, the study still aligns well with past research on the observation that the intensity of mental health service use of suicide decedents increases close to the date of suicide. The current study also shows what type of service contacts are negatively association with suicide (possibly indicating a protective effect, or a healthy user bias, as described in the study limitations below) and what type of service contacts are potential points of intervention for suicide prevention.

This study also used population level data from four public clinical mental health services datasets in Victoria; providing population level estimates of service use. This study is also novel in that it *combined* the data from these four main data sources to assess how various types of service contacts are associated with suicide.

### Limitations

All risk factors observed are from a mental health service system sample in this analysis; therefore, the interpretation cannot be applied to all people who die by suicide in the general population. Although not a limitation of the study itself, the study results describe patterns of service use and subsequent suicide *among public mental health service users in Victoria* and should therefore not be used for general interpretation of all suicides. Analysis of those who do not use mental health services but could benefit from doing so, in helping to prevent suicide, is beyond the scope of this study.

Healthy-user bias should be considered when interpreting the findings (26). Reported associations between certain treatment types and (lack of) suicide could in some cases be attributed to confounding factors. An underlying healthy behaviour could explain both the (non-occurrence of) suicide and the service use. Pro-active health behaviours can include treatment-seeking (and regularly turning up for appointments) as well as other healthy lifestyle choices, all of which can contribute to better outcomes. The current study cannot distinguish between the contributions of the various behaviours (as many relevant factors were not captured in the data): observational studies such as this one cannot accurately quantify the direct effect of service use on health outcomes. Therefore, it is not possible to establish a causal relationship between treatment and suicide prevention, in this observational study design.

Community contacts were negatively associated with suicide, suggesting a potential protective effect towards suicide. However, in this observational study, a causal association between these services and suicide prevention cannot be reliably established. Such research would require a randomized control trial.

## Conclusion

In a public mental health service user population, service use among suicide decedents is lower than among non-decedents. Non-decedents tend to have a stable level of service use over time, while suicide decedents tend to have more contact with health services proximal to suicide. An increasing number of mental-health related ED presentations and admissions to public hospital wards could be considered as intervention points for suicide prevention above the prevention mechanisms that are already in place. More community level contact could aid in monitoring patients that are likely to be at risk of suicide. The vast majority of people who died by suicide in Victoria were not accessing the clinical mental health services available through the public health system. A more general approach to suicide prevention may need to focus on access to, and uptake of, clinical public mental health services for those who need it.

## Data availability statement

The data analyzed in this study is subject to the following licenses/restrictions: The data used in this study are de-identified health service-use data and suicide data, both of which are considered confidential and sensitive. These data are available from the Centre for Victorian Data Linkage (CVDL), but restrictions apply to the availability of same, which were used under license for the current study, and so are not publicly available. The authors are not in a position to release the data (in accordance with the agreement with CVDL). Requests to access these datasets should be directed to CVDL@health.vic.gov.

## Ethics statement

This study was approved by the Monash University Human Research Ethics Committee (Project No. 14647). A waiver of informed consent was granted on the following grounds: the size of the linked datasets was large and obtaining consent from such a large number of persons was impracticable; the data were de-identified (name, date of birth, and other identifiers were removed from the dataset by the Centre of Victorian Data Linkage prior to the release of the data to the researchers); the study involvement was low risk for participants’ protection of privacy.

## Author contributions

JB-G and AC conceived the presented idea. TF conducted the analysis. MS provided the statistical guidance. AC provided the conceptual input. All authors were involved in developing the theory and interpreting the data, read, reviewed, revised, and approved the final manuscript.
