# Supplementary material for: Suicide among those who use mental health services: Suicide risk factors as evidenced from contact-based characteristics in Victoria
Source: Front Psychiatry. 2022 Dec 8;13:1047894. doi: 10.3389/fpsyt.2022.1047894 (PMC9772269; doi:10.3389/fpsyt.2022.1047894)
Supplement: Supplementary file 1 [file Data_Sheet_1.docx]

# Appendix 1

## Case selection

VSR data on all Coroner certified suicide deaths was supplied for the period 1 January 2012 to 31 December 2016.

### 12-month selection:

In all linked data sources, the analysis of contacts was limited to those that occurred in the 12 months prior to suicide death, using the date of death recorded in the VSR and dates of contact provided in other data sources.

#### VAED and CMI-ODS admissions

Hospital and residential admissions with an admission source indicating a transfer from another hospital or statistical separation (change in care type within the same hospital) in consecutive records were all considered to be part of the same episode.

### Fatal incident exclusion:

#### Hospital contacts

Any hospital (ED presentation, hospital admission) episode that ended in “death” was excluded as the episode was considered to be a result of the fatal incident (and therefore the episode was not an opportunity for suicide prevention).

## Data definitions

### Mental-health-related contact definitions

#### Mental-health-related admissions or ED presentations

Hospital admissions were considered to be mental-health-related if they were for reasons related to mental disorder, intentional self-harm and/or suicidal ideation.

- Mental disorder codes: any diagnosis code (1-40) in the range F00-F99
- Intentional self-harm: any diagnosis code (1-40) in the range X60-X84
- Suicidal ideation: any diagnosis codes (1-40) equal to R45.81

These categories are not mutually exclusive as more than one diagnosis code can be coded for one incident.

Hospital ED presentations were considered to be mental-health-related if they were for reasons related to mental disorder, intentional self-harm and/or suicidal ideation. Records with a primary diagnosis code for a general psychiatric examination or a history of self-harm were also included in the definition of mental-health-related ED presentations. (These codes are not used as principal diagnosis codes in the VAED).

- Mental disorder: any diagnosis code (1-3) in the range F00-F99
- Intentional self-harm: “human intent” variable equal to 2 (intentional self-harm)
- Suicidal ideation or emotional state: any diagnosis codes (1-3) equal to R45
- General psychiatric exam: primary diagnosis: primary diagnosis equal to Z046
- History of self-harm: primary diagnosis equal to Z915
- Departure status was transfer to a mental health bed at another hospital campus (17), returning to a mental health residential facility (23), ward setting at the same campus in a mental health observation/assessment unit (25) or other mental health bed at the same campus (26).
- Referred to a mental health community service, on departure (11)
- Referred by mental health telephone assessment/advisory line (16)

These categories are not mutually exclusive as more than one diagnosis code can be coded for one incident.

#### Overall definition of mental-health-related contact

Any record that met any of the following criterion was considered to be mental health related contact:

- Hospital admission or ED presentation for mental health,
- Any CMI admission
- Any CMI community contacts except the following program types (starting with D or M, A20, A22, E21, E22, E41, E42, E61, G30, K10, K11, K12, N30, N31, R20, R21, S01, S40, S41, S44, S49, S62, S81, T20, T30, DA22, DA32, DH22, E81, S70 and 99)
