# Supplementary material for: Suicide among those who use mental health services: Suicide risk factors as evidenced from contact-based characteristics in Victoria
Source: Front Psychiatry. 2022 Dec 8;13:1047894. doi: 10.3389/fpsyt.2022.1047894 (PMC9772269; doi:10.3389/fpsyt.2022.1047894)
Supplement: Supplementary file 2 [file Data_Sheet_2.docx]

*Table A1: Mean number of contacts by quarter and service type*

| Months before the index date | Cases | | Controls | | *p* value (comparing cases with controls by quarter) |
| --- | --- | --- | --- | --- | --- |
|  | Mean number of contacts (95% CI) | *p* value (compared with cases Q1) | Mean number of contacts (95% CI) | *p* value (compared with controls Q1) |  |
| Public hospital admissions |  |  |  |  |  |
| <=3 months relative to the index date (Q1) | 0.56 (0.50-0.63) | - | 0.18 (0.16-0.20) | - | <0.001 |
| 4-6 months relative to the index date (Q2) | 0.24 (0.18-0.29) | <0.001 | 0.19 (0.17-0.21) | 0.444 | 0.216 |
| 7-9 months relative to the index date (Q3) | 0.17 (0.13-0.22) | <0.001 | 0.20 (0.17-0.22) | 0.208 | 0.066 |
| 10-12 months relative to the index date (Q4) | 0.14 (0.11-0.18) | <0.001 | 0.19 (0.16-0.21) | 0.969 | 0.009 |
| ED presentations |  |  |  |  |  |
| <=3 months relative to the index date (Q1) | 0.77 (0.65-0.90) | - | 0.36 (0.31-0.41) | - | <0.001 |
| 4-6 months relative to the index date (Q2) | 0.36 (0.26-0.46) | <0.001 | 0.36 (0.32-0.41) | 0.891 | 1.000 |
| 7-9 months relative to the index date (Q3) | 0.24 (0.19-0.29) | <0.001 | 0.37 (0.32-0.41) | 0.689 | 0.008 |
| 10-12 months relative to the index date (Q4) | 0.20 (0.16-0.24) | <0.001 | 0.37 (0.32-0.42) | 0.536 | 0.001 |
| CMI/ODS residential admissions |  |  |  |  |  |
| <=3 months relative to the index date (Q1) | 0.03 (0.02-0.04) | - | 0.04 (0.03-0.04) | - | 0.229 |
| 4-6 months relative to the index date (Q2) | 0.02 (0.01-0.02) | 0.053 | 0.04 (0.03-0.04) | 0.630 | 0.001 |
| 7-9 months relative to the index date (Q3) | 0.01 (0.01-0.02) | 0.048 | 0.04 (0.04-0.05) | 0.066 | <0.001 |
| 10-12 months relative to the index date (Q4) | 0.01 (0.00-0.01) | 0.001 | 0.04 (0.03-0.04) | 0.635 | <0.001 |
| CMI/ODS community contacts |  |  |  |  |  |
| <=3 months relative to the index date (Q1) | 4.99 (4.37-5.62) | - | 15.25 (14.34-16.16) | - | <0.001 |
| 4-6 months relative to the index date (Q2) | 3.28 (2.73-3.82) | <0.001 | 15.95 (15.11-16.78) | 0.025 | <0.001 |
| 7-9 months relative to the index date (Q3) | 2.45 (2.06-2.84) | <0.001 | 16.50 (15.61-17.38) | 0.001 | <0.001 |
| 10-12 months relative to the index date (Q4) | 2.59 (2.14-3.04) | <0.001 | 16.85 (15.96-17.73) | <0.001 | <0.001 |

*Table A2: Odds of suicide death, 95% CI, and p value for the 12 months before the index date and by quarter before the index date*

| Characteristic | Overall (12 months) | |
| --- | --- | --- |
|  | OR (95% CI) | *p* value |
| Contact type (n) |  |  |
| Number of public hospital admissions | 1.04 (1.01-1.06) | 0.014 |
| Number of ED presentations | 1.08 (1.06-1.10) | 0.000 |
| Number of CMI/ODS residential admissions | 0.81 (0.65-1.01) | 0.064 |
| Number of CMI/ODS community contacts | 0.96 (0.96-0.96) | <0.001 |
|  |  |  |
| SEIFA |  |  |
| 1st quintile | 0.86 (0.66-1.13) | 0.285 |
| 2nd quintile | 1.12 (0.84-1.49) | 0.423 |
| 3rd quintile | 1.01 (0.79-1.29) | 0.936 |
| 4th quintile | 0.93 (0.76-1.14) | 0.501 |
| 5th quintile (reference) | - | - |
|  |  |  |
| Country of birth |  |  |
| Australia | 0.97 (0.81-1.18) | 0.788 |
| All other (reference) | - | - |
|  |  |  |
| Geographic region |  |  |
| Melbourne Metropolitan (reference) | - | - |
| Regional/Rural Victoria | 0.87 (0.70-1.08) | 0.202 |
|  |  |  |

*Table A2: contd…..*

| Characteristic | <=3 months relative to the index date (Q1) | | 4-6 months relative to the index date (Q2) | | 7-9 months relative to the index date (Q3) | | 10-12 months relative to the index date (Q4) | |
| --- | --- | --- | --- | --- | --- | --- | --- | --- |
|  | Quarter 1 | | Quarter 2 | | Quarter 3 | | Quarter 4 | |
|  | OR (95% CI) | *p* value | OR (95% CI) | *p* value | OR (95% CI) | *p* value | OR (95% CI) | *p* value |
| Contact type (n) |  |  |  |  |  |  |  |  |
| Number of public hospital admissions | 2.09 (1.82-2.40) | <0.001 | 1.25 (1.05-1.48) | 0.013 | 1.29 (1.03-1.62) | 0.026 | 1.35 (1.08-1.68) | 0.007 |
| Number of ED presentations | 1.13 (1.05-1.22) | 0.002 | 1.13 (1.00-1.28) | 0.048 | 1.02 (0.86-1.21) | 0.809 | 0.94 (0.81-1.08) | 0.389 |
| Number of CMI/ODS residential admissions | 0.77 (0.48-1.22) | 0.265 | 0.76 (0.42-1.37) | 0.367 | 0.78 (0.43-1.40) | 0.407 | 0.41 (0.18-0.91) | 0.028 |
| Number of CMI/ODS community contacts | 0.93 (0.92-0.94) | <0.001 | 0.90 (0.89-0.91) | <0.001 | 0.87 (0.86-0.88) | <0.001 | 0.88 (0.86-0.89) | <0.001 |
|  |  |  |  |  |  |  |  |  |
| SEIFA |  |  |  |  |  |  |  |  |
| 1st quintile | 0.79 (0.58-1.06) | 0.110 | 0.91 (0.69-1.20) | 0.496 | 0.88 (0.66-1.16) | 0.347 | 0.86 (0.65-1.13) | 0.281 |
| 2nd quintile | 0.95 (0.69-1.30) | 0.740 | 1.16 (0.87-1.54) | 0.322 | 1.06 (0.79-1.42) | 0.690 | 1.07 (0.80-1.44) | 0.645 |
| 3rd quintile | 1.05 (0.80-1.36) | 0.735 | 1.04 (0.81-1.33) | 0.752 | 0.99 (0.77-1.28) | 0.968 | 0.95 (0.74-1.22) | 0.693 |
| 4th quintile | 0.94 (0.76-1.17) | 0.595 | 0.93 (0.76-1.15) | 0.510 | 0.95 (0.77-1.17) | 0.613 | 0.87 (0.71-1.07) | 0.176 |
| 5th quintile (reference) | - | - | - | - | - | - | - | - |
|  |  |  |  |  |  |  |  |  |
| Country of birth |  |  |  |  |  |  |  |  |
| Australia | 0.93 (0.76-1.14) | 0.474 | 1.02 (0.84-1.23) | 0.836 | 1.00 (0.82-1.21) | 0.992 | 1.00 (0.82-1.21) | 0.960 |
| All other (reference) | - | - | - | - | - | - | - | - |
|  |  |  |  |  |  |  |  |  |
| Geographic region |  |  |  |  |  |  |  |  |
| Melbourne Metropolitan (reference) | - | - | - | - | - | - | - | - |
| Regional/Rural Victoria | 1.07 (0.85-1.35) | 0.540 | 0.94 (0.76-1.17) | 0.597 | 0.91 (0.73-1.13) | 0.403 | 0.89 (0.71-1.11) | 0.297 |
